# Affordable 3D Orientation Visualization Solution for Working Class Remotely Operated Vehicles (ROV)

**DOI:** 10.3390/s24165097

**Published:** 2024-08-06

**Authors:** Mohammad Afif Kasno, Izzat Nadzmi Yahaya, Jin-Woo Jung

**Affiliations:** 1 Department of Computer Science and Engineering, Dongguk University, Seoul 04620, Republic of Korea; mohammad.afif@utem.edu.my; 2 Faculty of Electronic Technology and Engineering, Universiti Teknikal Malaysia Melaka, Malacca 76100, Malaysia

**Keywords:** working class ROV, cost-effective ROV 3D visualization, 3D ROV orientation awareness, real-time visualization, working class ROV operational constraints

## Abstract

ROV operators often encounter challenges with orientation awareness while operating underwater, primarily due to relying solely on 2D camera feeds to manually control the ROV robot arm. This limitation in underwater visibility and orientation awareness, as observed among Malaysian ROV operators, can compromise the accuracy of arm placement, and pose a risk of tool damage if not handle with care. To address this, a 3D orientation monitoring system for ROVs has been developed, leveraging measurement sensors with nine degrees of freedom (DOF). These sensors capture crucial parameters such as roll, pitch, yaw, and heading, providing real-time data on the ROV’s position along the X, Y, and Z axes to ensure precise orientation. These data are then utilized to generate and process 3D imaging and develop a corresponding 3D model of the operational ROV underwater, accurately reflecting its orientation in a visual representation by using an open-source platform. Due to constraints set by an agreement with the working class ROV operators, only short-term tests (up to 1 min) could be performed at the dockyard. A video demonstration of a working class ROV replica moving and reflecting in a 3D simulation in real-time was also presented. Despite these limitations, our findings demonstrate the feasibility and potential of a cost-effective 3D orientation visualization system for working class ROVs. With mean absolute error (MAE) error less than 2%, the results align with the performance expectations of the actual working ROV.

## 1. Introduction

Since their introduction in the 1960s, remotely operated vehicles (ROVs) have become indispensable tools in underwater research and intervention. These robotic systems have significantly advanced our knowledge of the deep ocean, facilitating scientific exploration, industrial activities, and environmental surveillance in the most challenging and remote underwater environments, and navigating the abyssal depths which are beyond human reach [1,2,3,4].

The evolution from traditional 2D to advanced 3D visualization technologies marks a significant milestone in the history of ROVs. Initial models, equipped with monocular 2D camera systems, provided vital visuals of the submerged environments but were inherently limited. The lack of depth perception and spatial awareness posed substantial challenges in navigation, manipulation, and data collection within the complex, often unpredictable marine landscapes [5,6,7,8].

Despite these advancements, maintaining precise orientation awareness remains a formidable challenge for ROV operators. The inherent limitations of even the most sophisticated 2D systems can obscure the ROV’s orientation, complicating the use of onboard tools and potentially jeopardizing mission success. Environmental factors, such as turbulent currents and unexpected encounters with marine life, can further disrupt the ROV’s positioning, often without the operator’s knowledge, introducing risks of mis navigation or damage to the vehicle and its surroundings [9].

Prior studies have endeavored to address these issues. According to M.S.M. Aras, the principal aim of their project is to refine the navigation of unmanned underwater vehicles (UUVs) using a cost-effective combination of the accelerometer ADXL-345 sensor (Analog Devices, Norwood, MA, USA), and gyroscope ITG-3200 sensor (InvenSense, San Jose, CA, USA). By leveraging the data output from this sensing unit (IMU), which includes angular rate, degree, and translational acceleration, improvements can be made to UUV navigation functionalities such as auto depth control, lateral movement, and obstacle avoidance. Enhancing these aspects typically involves modifying algorithms, a process made easier and less time-consuming with access to output graphs and 3D real-time animations. Such a system can enable UUVs to effectively explore sea depths, conduct surveys, and tackle various challenges. The primary objective of the project is to develop an inertial sensing unit comprising the accelerometer ADXL-345 sensor and gyroscope ITG-3200 sensor to capture information about UUV angular rate, acceleration, and movement. Ensuring the sealing or airtightness of the sensing device is crucial for its performance and safety [10,11].

Image-based visual servoing (IBVS) has tackled the issue of depth perception in underwater robotic applications by executing control tasks in the 2D image plane instead of using 3D model-based control methods. Karras and colleagues (2022) proposed a new IBVS control approach for floating base mobile manipulator systems (FBMMSs), ensuring predetermined performance qualities and tackling visibility limitations from the camera’s restricted field of vision. This technique is resilient to uncertainties in the environment and disturbances, which is essential for ensuring stable operations in chaotic underwater settings [12].

Additional progress involves the implementation of control barrier functions (CBFs) for secure force/position tracking in FBMMSs, as shown by Sharifi et al. (2024). This method addresses crucial safety factors like manipulator joint limits and system velocity constraints, while also being resilient to uncertainties in system dynamics [13]. Moreover, event-based predictive control techniques have demonstrated potential in improving the speed and effectiveness of visual servoing systems. Research conducted by Aspragkathos and colleagues (2024) introduces a predictive control framework for UAVs based on event-triggered image moments, emphasizing its potential use for underwater vehicles [14]. Heshmati-Alamdari and colleagues (2023) present a visual servoing technique designed for underwater vehicle manipulator systems which combines predictive control and CBFs to guarantee safety and performance while adhering to operational limitations [15].

In the field of underwater robotics, many commercial software options offer advanced real-time 3D visualization features for ROV tasks. Fledermaus provides robust 4D geospatial analysis and visualization features, merging sonar and laser scanner data to produce intricate 3D maps of underwater settings [16]. Likewise, EIVA NaviSuite is a full package created for marine surveying and ROV operation, allowing users to navigate and examine underwater structures accurately by connecting sonar, cameras, and various sensors [17].

However, the challenges posed by advanced ROV technologies, such as economic and accessibility barriers, continue to be significant hurdles. The expensive nature of developing, deploying, and maintaining advanced underwater robotics systems makes them inaccessible for many, hampering their wider use and innovation. The intricate nature of these systems also demands specific expertise and education, further limiting the availability to only a small group of operators and researchers [12].

Our objective is to create an affordable 3D orientation display system for ROVs used by the working class. This system uses affordable nine degrees of freedom sensors and open-source integration platforms to deliver accurate real-time 3D orientation data, ensuring precise navigation and operation in challenging underwater conditions. Nevertheless, the experimental data are restricted by constraints established in an agreement between the researchers and the ROV operators. Our study was carried out with the restriction that only brief tests (lasting up to 1 min for each of three positions) could be executed under controlled conditions. This limitation exists because operators must keep their operations secret and face difficulties in obtaining managerial approval for long testing periods. Despite these drawbacks, we think our research adds value by showing the possibility and promise of an affordable 3D orientation visualization system for ROVs used by working class ROV operators. This approach addresses the technical and operational limitations present in current systems, as well as eliminating economic and accessibility barriers, enabling a greater number of individuals to utilize sophisticated orientation awareness technology.

To achieve a good level of accuracy and reliability in our affordable 3D orientation visualization system, we incorporate filtering techniques to process the data obtained from the sensors. One of the key challenges in accurately interpreting sensor data is the presence of noise, which can significantly affect the performance and precision of orientation measurements. 

## 2. Kalman Noise Filter Algorithm

Given the critical role of clean and accurate data in our system, it is necessary to apply filtering techniques because raw data from IMU sensors often contain noise resulting from physical vibrations, particularly in the case of accelerometers, while gyroscopes tend to exhibit drift over time [18,19].

Although there are more advanced noise filters (like the extended Kalman filter with bias consideration for 9-DOF IMU-based attitude and heading estimation by Farahan S.B. et al. [20], or the complementary Kalman filter used for multi-rotor drones stabilization in [21], smoothed error state Kalman filter for sensor fusion of GNSS and IMU data in [22]) that offer enhanced handling of errors from drift over time, our current work employs a basic Kalman noise filter due to experimental setup limitations and the absence of significant drifting issues. We plan to explore these advanced noise filtering techniques in future work.

The IMU signals readable in Arduino are initially in raw data units and are filtered using a Kalman noise filter. The filter aims to estimate the true state of the sensor, such as orientation or velocity from noisy measurements obtained from accelerometer, gyroscope, and magnetometer using GY-85. The Kalman filter equations can be calculated below:

(1) $$K_{k}={P_{k|k-1}H_{k}^{T}\left(H_{k}P_{k|k-1}H_{k}^{T}+R_{k}\right)}^{-1}$$

$K_{k}$ is the Kalman gain, balancing the importance of the new measurement $z_{k}$ against the predicted state. $H_{k}$ maps the state to the measurement space, and $R_{k}$ represents the measurement noise.

(2) $${\hat{x}}_{k|k-1}=F_{k}{\hat{x}}_{k-1|k-1}+B_{k}u_{k}$$

${\hat{x}}_{k|k-1}$ is the predicted state before the new measurement at time *k*, using the system’s dynamics $F_{k}$ and control input $B_{k}u_{k}$. It is a prediction model which takes both the natural evolution of the system over time and any external influences via control inputs.

(3) $${\hat{x}}_{k|k}={\hat{x}}_{k|k-1}+K_{k}\left(z_{k}-H_{k}{\hat{x}}_{k|k-1}\right)$$

${\hat{x}}_{k|k}$ is the updated state and combines the predicted state with the new measurement $z_{k}$, adjusted by the Kalman gain.

(4) $$P_{k|k-1}=F_{k}P_{k-1|k-1}F_{k}^{T}+Q_{k}$$

$P_{k|k-1}$ is the estimated uncertainty (covariance) of the state before the new measurement, and $Q_{k}$ is the process noise, indicating how much noise is expected in the system’s dynamics.

(5) $$P_{k|k}=\left(I-K_{k}H_{k}\right)P_{k|k-1}$$

where $P_{k|k}$ is the updated covariance and reflects the reduced uncertainty after incorporating the new measurement. 

## 3. Inertial Measurement Unit (IMU) Algorithm

The IMU raw data signals that are readable in Arduino are filtered using the Kalman noise filter mentioned above. They are then transformed into acceleration and angular velocity to plot the output graph [10,15]. To obtain the required output units, several fundamental equations must be applied, where *X*, *Y*, and *Z* denote the raw data from the sensor to the microcontroller. Subsequently, utilizing the Pythagorean theorem, the distance between *X*, *Y*, and *Z* can be calculated:

(6) $$X=\sqrt{Y^{2}+Z^{2}}$$

(7) $$Y=\sqrt{Z^{2}+X^{2}}$$

(8) $$Z=\sqrt{X^{2}+Y^{2}}$$

Using trigonometric formulas, the angles can be computed from Equations (6)–(8) as below:

(9) $$\theta_{x}=\alpha rctan\left(\frac{x_{1}}{x_{2}}\right)$$

(10) $$\theta_{y}=\alpha rctan\left(\frac{y_{1}}{y_{2}}\right)$$

(11) $$\theta_{z}=\alpha rctan\left(\frac{z_{1}}{z_{2}}\right)$$

The angles relative to the *X*-axis, *Y*-axis, and *Z*-axis can be established based on Equations (9)–(11). To convert the radian values from *X*, *Y*, and *Z* to degrees, Equation (12) is utilized.

(12) $$X^{\left(o\right)}=\frac{\theta_{x}\times 180}{\pi }$$

**Figure 1:**
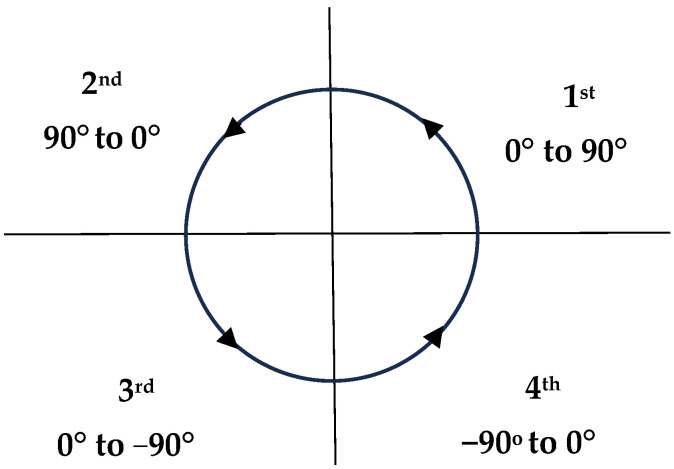
Orientation of Euler angle output at *X*-axis.

The IMU does not output degrees ranging from 0 to 360 for a complete circle. Instead, for the *X*-axis and *Y*-axis, the first quadrant spans from 0 to 90 degrees, the second quadrant from 90 to 0 degrees, the third quadrant from 0 to −90 degrees, and the fourth quadrant from −90 to 0 degrees, as illustrated in Figure 1. This is typical for many IMUs, which use Euler angles instead of the 2D Cartesian coordinate system to represent orientation. IMUs typically have their orientation angles limited to a range of −180 degrees to 180 degrees (or −π to π radians) because trigonometric functions such as arctangent are utilized in angle calculations. These functions loop back around at these boundaries because they repeat regularly. Similarly, for the *Z*-axis, the first quadrant ranges from 0 to 90 degrees, the second quadrant from −90 to 0 degrees, the third quadrant from 0 to −90 degrees, and the fourth quadrant from 90 to 0 degrees.

## 4. Proposed 3D Orientation Visualization for Working ROV

The 3D orientation visualization is created using the GY 85 IMU sensor (Kuongshun Electronic, Shenzhen, China), which offers nine degrees of freedom (DOF) sensor data. This includes a three-axis gyroscope, triaxial accelerometer, and three-axis magnetic field readings. Communication is established with the Magnetometer HMC5883L module (Honeywell, Charlotte, NC, USA), Triple Axis gyroscope ITG3205 module (InvenSense, San Jose, CA, USA), and accelerometer ADXL345 module (Analog Devices, Norwood, MA, USA) via an I2C interface.

**Figure 2:**
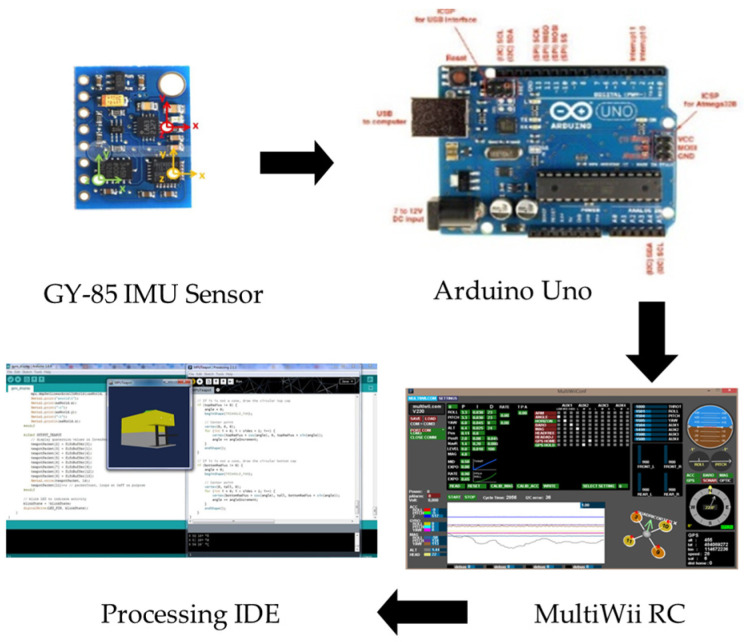
Hardware and software setup using GY-85 IMU sensor, Arduino Uno, MultiWii RC and Processing IDE.

The data from the IMU sensor is initially processed using an Arduino Uno (Cytron Technologies, Penang, Malaysia), where algorithms and calculations are applied. These processed values are subsequently transmitted to MultiWii 2.4 software, an open-source autopilot commonly utilized by researchers, as noted by [23], for real-time graph representation of the IMU sensor values. The data are then converted to the Processing IDE 4.3 to animate the 3D model according to the received values. The development of the 3D model is based on reference to the operational ROV used by Malaysian operators in the working class. Figure 2 illustrates the setup process.

**Figure 3:**
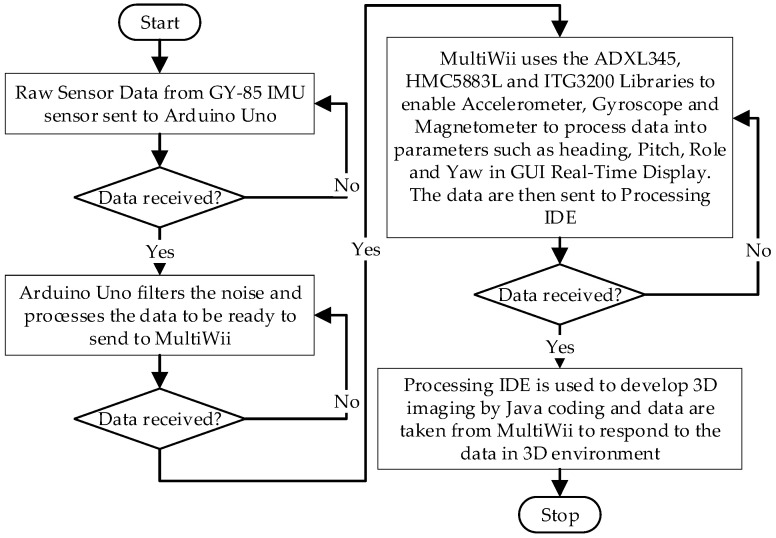
Flowchart for the proposed methodology.

Figure 3 elucidates the programming procedure outlined in the proposed methodology. The raw sensor data captured by the GY-85 IMU sensor is seamlessly transmitted to the Arduino Uno in real-time via I2C interfacing. The communication operates at a baud rate of 115,200 for monitoring through the serial monitor. Subsequently, the Arduino Uno initiates noise filtering and data processing to prepare the information for transmission to MultiWii. The details pseudo-code interfacing are explained in Appendix A. 

## 5. Experimental Results

### 5.1. Real-Time Reading in MultiWii

**Figure 4:**
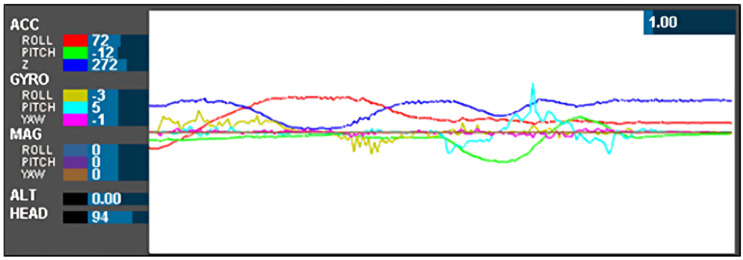
The readings of roll, pitch, and yaw in MultiWii.

Figure 4 shows the representation of roll, pitch, and yaw in graphical interpretation. MultiWii is a general-purpose software with built-in libraries for a multirotor RC model. MultiWii has an extension available for integrated ADC up to 14-bit analog–digital converter. This extension communicates with the controller on an I2C bus in fast mode at 400 kbit/s. MultiWii uses orientation libraries to enable Accelerometer, Gyroscope, and Magnetometer to process the filtered data into parameters such as heading, pitch, roll, and yaw in the graphic user interface (GUI) real-time display [24].

**Figure 5:**
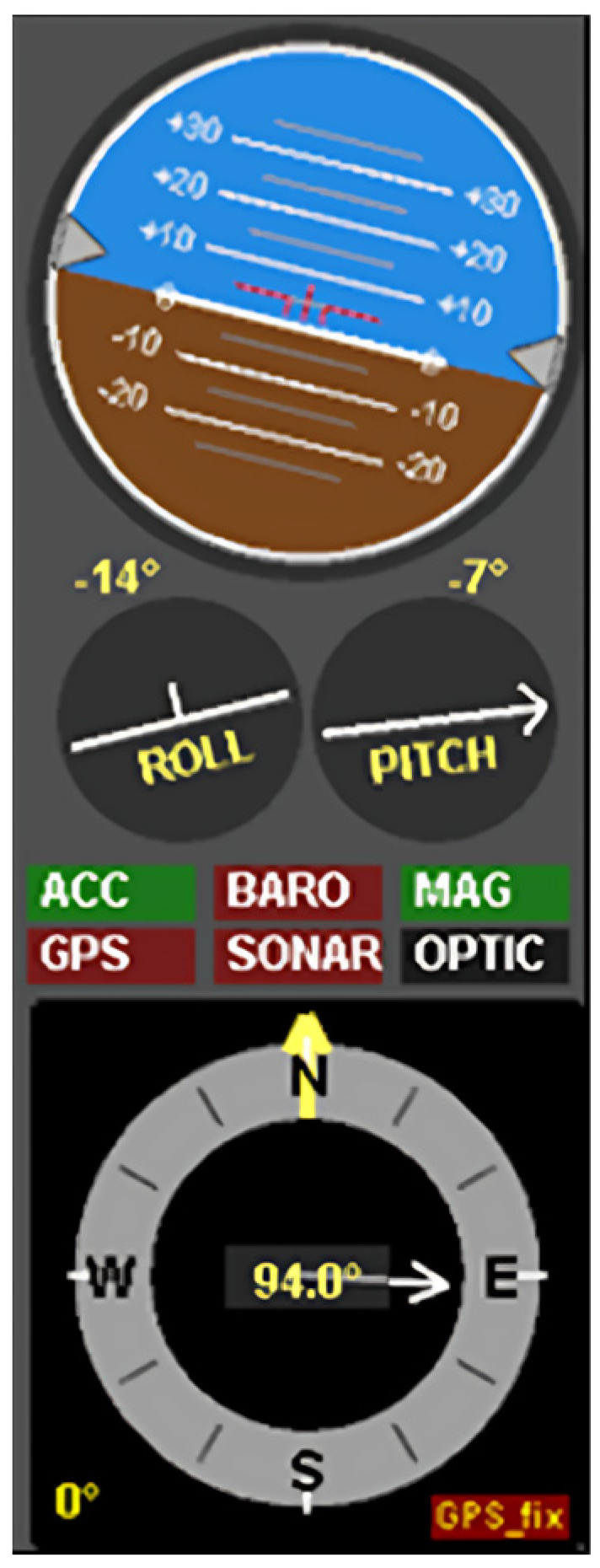
The GUI of roll, pitch, and yaw in MultiWii.

Subsequently, the data is transmitted to the Processing IDE software for real-time 3D modeling response. Figure 5 displays the graphical user interface (GUI) featuring roll, pitch, and yaw parameters. The GUI depicted in both figures indicates that the real-time data presentation is effective and responsive. Moreover, it can be adapted for operators who prefer a meter-style GUI interface, resembling the typical interface seen in ROV cabin controllers. 

### 5.2. Three-Dimensional Modeling in Processing IDE

The 3D modeling representing the working class ROV has been pre-programmed in Java using the Processing IDE. Processing IDE, an open-source software, facilitates the display of 3D models and GUI elements through its graphical libraries [17]. Consequently, the data received from MultiWii are seamlessly integrated into the 3D modeling process to provide real-time orientation visualization. In Figure 5, the front view of the actual ROV is depicted alongside the 3D modeling from various angles. The 3D modeling adheres to the structural design of the working ROV, as per the specifications requested by local operators, to ensure easy recognition of orientation during underwater operations.

The 3D image of the ROV was programmed to maintain a similar ratio to the actual ROV’s structure. However, as the 3D image primarily represents the basic structure, intricate details such as electrical and mechanical components (e.g., fan, engine, robot arm, wiring) were not included. Consequently, while the design of the 3D image provides a general representation, it does not precisely match the actual ROV. Additionally, the 3D image does not account for environmental factors such as underwater pressure, resistance, buoyancy, sea currents, or the ROV’s total mass. Therefore, it does not accurately simulate the real-world conditions experienced by the ROV.

The 3D image was crafted without factoring in these environmental variables, as its primary objective is to simulate the orientation of the ROV. Another notable distinction lies in the overall design of edges: while the 3D image features sharp edges, the actual ROV possesses smoother edges. Additionally, in the 3D image, the robot arm is depicted as fixed, as the monitoring system does not incorporate orientation tracking for the robot arm. In contrast, in real working-class ROVs, operators utilize various types of robot arms tailored to specific subsea tasks, such as pipe cutting or welding, each requiring different settings. Hence, operators adjust the robot arm configuration based on the tasks assigned for each dive. Furthermore, including the robot arm in the 3D design may not be essential, as the primary purpose of the 3D model is to monitor the ROV’s body. However, in the actual ROV, careful consideration is given to the design and functionality of the robot arm, as it serves a critical role in various underwater operations.

**Figure 6:**
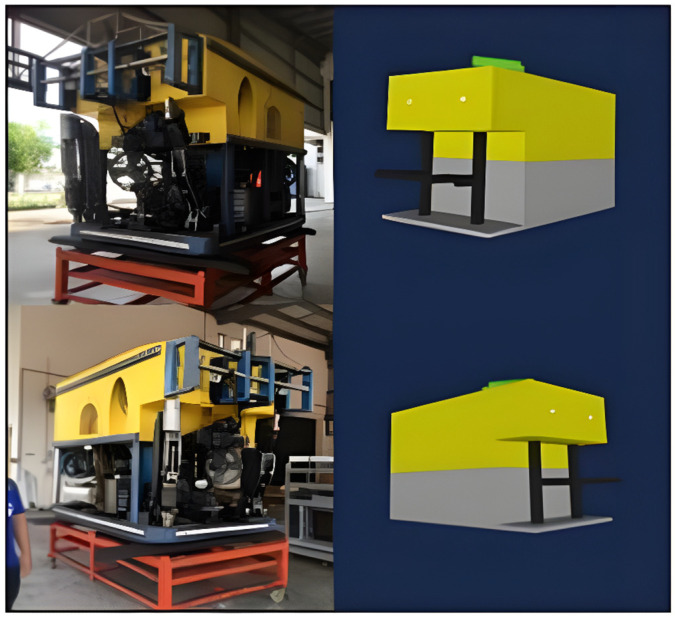
Top-left image: Front view of the actual working class remotely operated vehicle (ROV) used by Malaysian operators. Bottom-left image: Another angle of the actual ROV. Top-right image: The 3D model of the ROV as developed in our study, which replicates the front view of the actual ROV. Bottom-right image: An alternate perspective of the 3D model.

**Figure 7:**
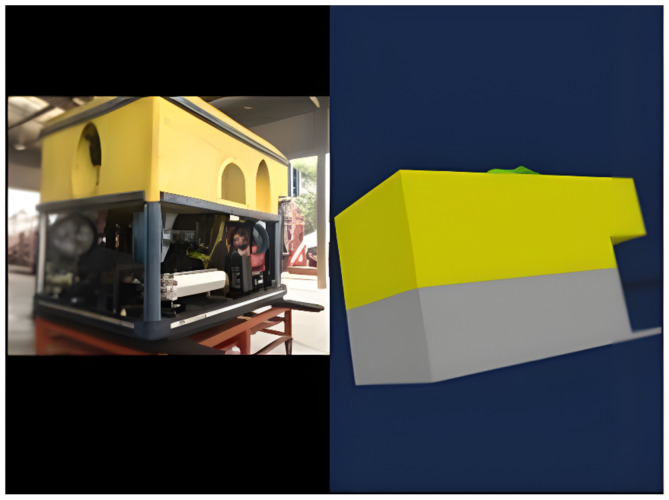
Left image: Back view of the actual ROV. Right image: Back view of the 3D model.

Subsequently, a directional arrow was incorporated into the 3D image to indicate the ROV’s point of view, facilitating differentiation between the front and back views within the simulator. To enhance the resemblance to a real working ROV, headlamps were integrated into the design. Figure 6 and Figure 7 present a comparative analysis between the actual ROV and the 3D image rendering.

### 5.3. GY-85 IMU Sensor Testing

Due to potential errors in raw IMU sensor measurements, especially offset readings, leading to integration errors and eventual drifting of attitude and position estimates, IMUs are typically reliable for brief measurement periods, often lasting only seconds [25] or up to a few minutes. The precision of extended measurement trials is significantly impacted by the changing motion conditions tracked [26]. Also, due to the limitation of experimental setup with the working ROV, we ran the experimentation assessment in idle position, 45 degrees to the right, and 45 degrees to the left in 1 min duration data capture.

**Figure 8:**
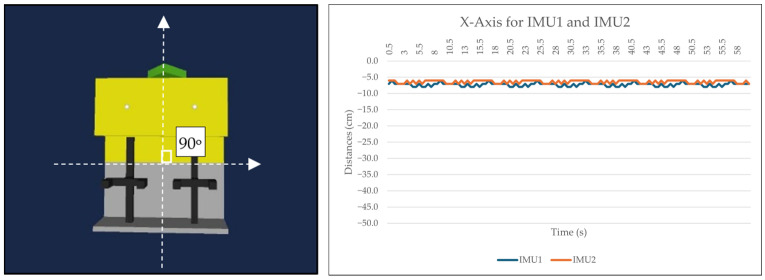
Data and ROV orientation while in idle *X*-axis position.

**Figure 9:**
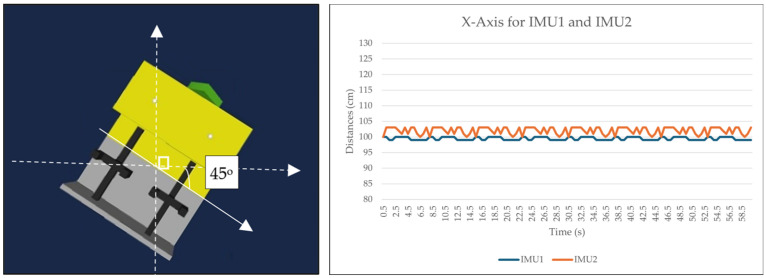
Data and ROV orientation of *X*-axis rotated 45 degrees to the right.

**Figure 10:**
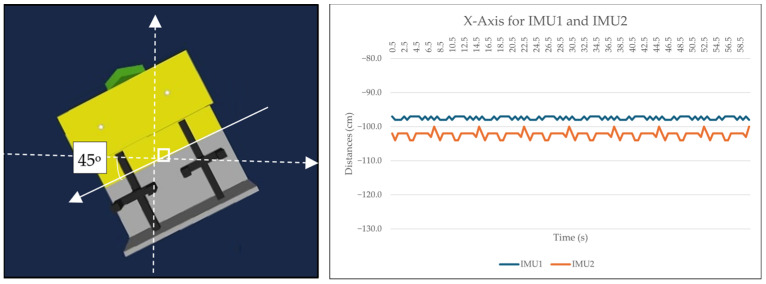
Data and ROV orientation of *X*-axis when rotated 45 degrees to the left.

Two 9-DOF IMU GY-85 sensors were employed to assess the accuracy and latency between them. Three sets of data were collected for the *X*-axis (roll), corresponding to different conditions: idle state, positive value (turning right by 45 degrees), and negative value (turning left by 45 degrees). The IMU sensors were positioned at the midpoint of the ROV. Data were sampled at intervals of 0.5 s for each condition over the duration of 1 min. Figure 8, Figure 9 and Figure 10 depict the collected data presented graphically alongside the 3D orientation position of the ROV. It should be noted that variations in the data may occur with different setups. In our experimental setup both IMU sensors were situated near the center of the actual working class ROV and moved within a shipyard environment rather than being submerged in the subsea.

Both IMU sensors (IMU1 and IMU2) were simultaneously recorded under three different conditions (idle, 45 degrees to the left, 45 degrees to the right). An Arduino Uno was connected to IMU1, while an Arduino MEGA 2560 (Cytron Technologies, Penang, Malaysia) was connected to IMU2. Data were sampled every 0.5 s. The initial analysis focused on the *X*-axis in the idle position, with the sensor calibrated to zero degrees. Figure 8 illustrates that both IMU sensors maintained similar *X*-axis values between 2.5 and 3.5 s throughout the one-minute duration. Although slight fluctuations were observed in some values, overall stability was maintained. The second analysis involved turning the IMU sensor to the right by 45 degrees. Figure 9 depicts that while IMU1 remained consistently stable, IMU2 exhibited minor fluctuations during the 3.5 s to 4.5 s interval over the course of one minute, possibly influenced by environmental factors. However, these changes were within acceptable tolerance limits. In the third analysis, the sensor was rotated 45 degrees to the left. Both IMU1 and IMU2 exhibited marginal changes while remaining stable, as shown in Figure 10. The *X*-axis data presented are raw data extracted from sketches programmed in the Arduino IDE.

**Figure 11:**
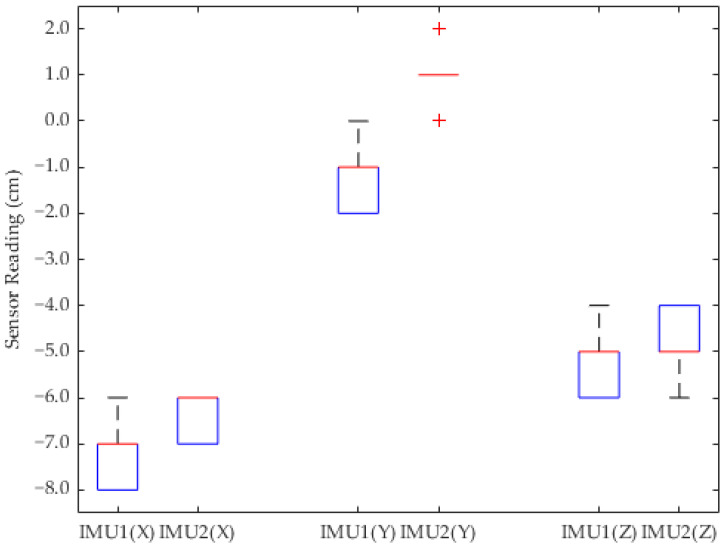
Boxplot of *X*, *Y*, and *Z* axis sensor readings in idle position.

**Figure 12:**
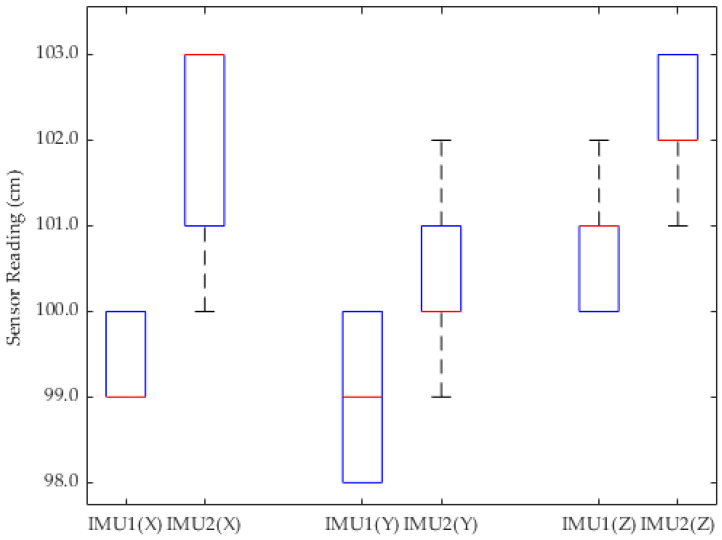
Boxplot of *X*, *Y*, and *Z* axis sensor readings in 45 degrees to the right position.

**Figure 13:**
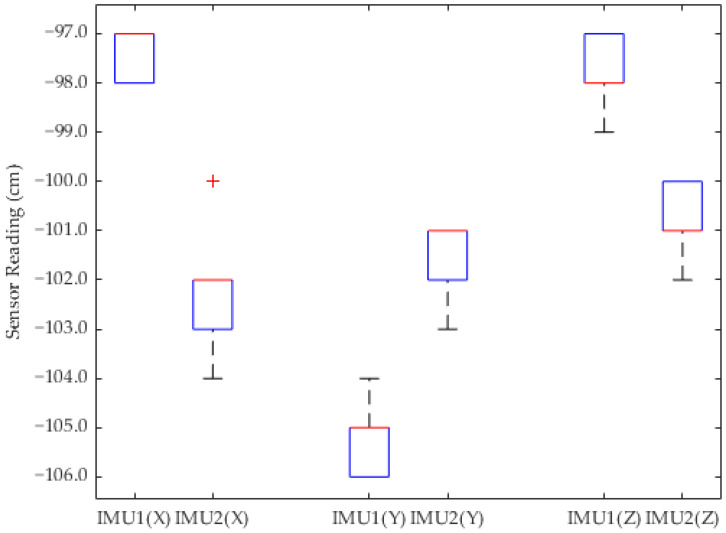
Boxplot of *X*, *Y*, and *Z* axis sensor readings in 45 degrees to the left position.

Referring to Figure 11, Figure 12 and Figure 13, the boxplot analysis of GY-85 IMU sensor readings for conditions including idle, 45 degrees left, and 45 degrees right orientations, serves to identify the central tendency, dispersion, and outliers within the collected data. Each boxplot represents the distribution of sensor readings across these predefined orientations, illustrating the median (central line), the interquartile range (IQR, the box), and potential outliers. The median value is represented by the central line with red color in every box. The first and third quartiles (Q1 and Q3) are represented by the edges of the box, showing the interquartile range (IQR). The whiskers stretch to the furthest points that are 1.5 times the IQR away from the quartiles. Outliers are indicated by "+" signs and represent sensor readings outside the whiskers. Blue lines represent data from IMU1 while red lines represent data from IMU2.

The consistency in the median values across different conditions indicates the sensor’s reliability in detecting orientation changes. The IQR provides insights into the variability of the sensor readings, where a narrow IQR suggests a high level of precision. Observing only minimal outliers, or even none, further underscores the sensors’ accuracy and robustness against random errors or environmental interferences.

Despite encountering fluctuation of data in the sensor readings, the overall analysis suggests these variations are negligible due to the very small gap (in centimeters) and not highly accurate or sensitive applications. This reinforces the premise that GY-85 sensors are sufficiently accurate for generating reliable 3D orientation visualizations for ROVs, supporting operational decision making, and enhancing navigation and manipulation capabilities in underwater environments.

**Figure 14:**
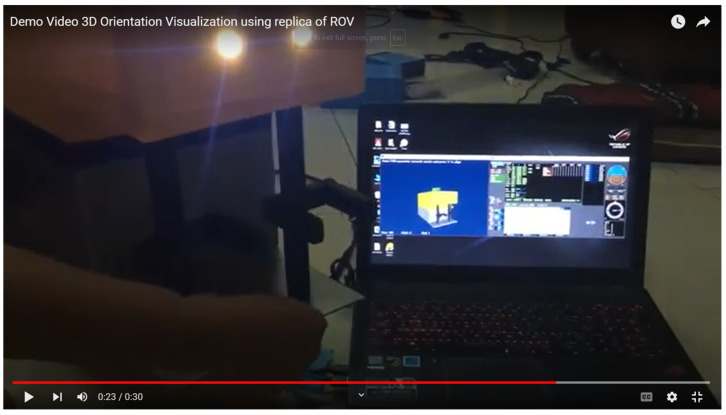
Screenshot of the replica demonstration video.

To further enhance the understanding and novelty of our project, we created a video demonstration that shows the replica of the working ROV moving and reflecting in the 3D simulation in real-time (see Supplementary Materials). This video provides a visual representation of the system’s functionality and effectiveness. Figure 14 shows a screenshot of the replica demonstration.

The overall comparison (Table 1) for IMUs in idle position, and tilted 45 degrees left and right, provides a straightforward assessment of IMU performance across various scenarios. This is the essence of the analysis:

During the idle position, the mean absolute error (MAE) values for the *X*, *Y*, and *Z* axes are 0.842, 2.192, and 0.783, resulting in error percentages of 0.234%, 0.609%, and 0.218%, respectively. The values are significantly below 1%, suggesting a minor difference that may not be a concern for applications that do not require high precision. Placing the IMUs at a 45-degree angle to the left results in a small increase in MAE values, with measurements of 5.4, 3.8, and 3.4 for the *X*, *Y*, and *Z* axes, respectively. These values correspond to 1.50%, 1.06%, and 0.94% of the complete range which assuming the full range of motion for each axis is ±180 degrees. Even though the percentage errors are higher than at the idle position, they still stay below 2%. In the same way, for the condition where the angle is 45 degrees to the right, the MAE values are 2.533, 1.400, and 1.533, each with percentage errors of 0.704%, 0.389%, and 0.426%, accordingly. Like the other two situations, these mistakes account for less than 1% of the total range, thus supporting the assertion that the accuracy of the IMUs is sufficient for ROV 3D visualization.

To sum up, the errors in all scenarios and directions are small enough to determine that the IMUs perform consistently and dependably for the task of ROV orientation in three-dimensional space. The small error rates show that the IMU readings are highly accurate, suggesting that for the non-essential precision needs of 3D visualization for ROVs, the IMU errors can be seen as insignificant and typically overlooked. This makes it possible to use the data confidently without relying on complicated error correction algorithms, making the process simpler and ensuring efficiency in visualization tasks.

## 6. Conclusions

This research has presented and assessed a new 3D orientation display system for working class remotely operated vehicles (ROVs), utilizing the features of GY-85 inertial measurement unit (IMU) sensors. This system focuses on the important problem of underwater orientation awareness, a significant concern for ROV operators who usually depend on 2D camera feeds. Our system suggests operational efficiency and safety by providing a real-time 3D orientation display system for working class ROVs. The GY-85 sensors have been shown in our experiments to be both reliable and accurate in capturing the required orientation parameters. Although there were slight changes in sensor data during different testing scenarios, such as stationary, 45 degrees left, and 45 degrees right positions, the findings show that these differences are acceptable for the intended use. The box-plot examination emphasizes the sensors’ steady performance, showing a narrow IQR and few outliers, indicating the system’s precision. The small value of MAE percentage from the comparison table where less than 1% error for idle position, less than 2% error for 45 degrees to the left and less than 1% error for 45 degrees to the right also shows that the errors are considered negligible and generally ignored.

Nonetheless, our ability to collect experimental data is restricted to brief tests (maximum of 1 min) because of the restrictions imposed by the agreement with ROV operators. This restriction hinders our capacity to adequately tackle possible long-term problems like drift and error buildup. Subsequent studies will include longer testing periods to thoroughly assess these factors. Furthermore, incorporating extra sensor information like sonar or stereo cameras might improve the 3D surroundings and offer superior navigation points. Even with these restrictions, our study emphasizes features, such as incorporating inexpensive sensors, employing noise filtering methods, and achieving real-time 3D visualization with open-source platforms for working class ROV. We suggest additional enhancements and new research paths to improve the system’s capabilities and reliability.

In summary, this research confirms the efficiency of utilizing GY-85 IMU sensors for 3D orientation display in ROVs. Our system provides an affordable, precise, and user-friendly answer to the long-standing issues with underwater navigation awareness for economic constraints of ROV operators, paving the way for new opportunities in deep-sea research, exploration, and operations. In the future, efforts will be directed towards improving the precision of the system with experiments of drifting in real navigation, investigating the incorporation of more sensor technologies such as IBVS, and broadening its usage to various underwater vehicles and situations.

## Figures and Tables

**Table 1:** Mean Absolute Error for the measurements.

Axis	Mean IMU1	Mean IMU2	Standard Deviation COM 3	Standard Deviation COM 6	Mean Absolute Error (MAE)	Percentage of Full Range (%)
Idle Position						
*X*-Axis	−7.225	−6.383	0.557	0.488	0.842	0.234
*Y*-Axis	−1.258	0.933	0.458	0.444	2.192	0.609
*Z*-Axis	−5.075	−4.725	0.700	0.661	0.783	0.218
45 Degrees to the Left						
*X*-Axis	−97.4	−102.8	0.55	1.10	5.4	1.50
*Y*-Axis	−105.4	−101.6	0.55	0.55	3.8	1.06
*Z*-Axis	−97.8	−101.2	0.84	0.84	3.4	0.94
45 Degrees to the Right						
*X*-Axis	99.467	102.0	0.501	1.160	2.533	0.704
*Y*-Axis	98.867	100.267	0.809	0.775	1.400	0.389
*Z*-Axis	100.80	102.20	0.751	0.751	1.533	0.426

## Data Availability

Data will be made available upon request.

## Supplementary Materials

A video demonstration of the working ROV moving and reflecting in the 3D simulation in real-time can be viewed at the following link: https://youtu.be/X7P5N5nv39k (accessed on 27 June 2024).

## Appendix A. Pseudo-Code of Interfacing Implementation

Below is the batch of pseudo-codes for the proposed method.

**Algorithm A1** GY85 IMU Data Retrieval Procedure
**Input:** GY85—GY85 IMU sensor object. **Output:** *sensorData*—A structure containing raw sensor data from GY85 IMU **Procedure** getRawSensorDataFromGY85IMU: 1 *ax* ← GY85.accelerometer_x (GY85.readFromAccelerometer()) 2 *ay* ← GY85.accelerometer_y (GY85.readFromAccelerometer()) 3 *az* ← GY85.accelerometer_z (GY85.readFromAccelerometer()) 4 *cx* ← GY85.compass_x (GY85.readFromCompass()) 5 *cy* ← GY85.compass_y (GY85.readFromCompass()) 6 *cz* ← GY85.compass_z (GY85.readFromCompass()) 7 *gx* ← GY85.gyro_x (GY85.readGyro()) 8 *gy* ← GY85.gyro_y (GY85.readGyro()) 9 *gz* ← GY85.gyro_z (GY85.readGyro()) 10 **Return** a structure containing *ax*, *ay*, *az*, *cx*, *cy*, *cz*, *gx*, *gy*, *gz* **Begin** Data Retrieval Procedure *sensorData* ← getRawSensorDataFromGY85IMU() **End** Data Retrieval Procedure

With the GY85 IMU Sensor object as input and *sensorData* as output, the collection of raw data from IMU sensors by variables named *ax*, *ay*, and *az* for accelerometer; *cx*, *cy*, and *cz* for compass; and *gx*, *gy*, and *gz* for gyro sensor. The collected data are returned as a single structured entity that encapsulates all the individual sensor readings. The collected data will then pass to the noise filtering process using Kalman filter as below:

**Algorithm A2** Noise Filtering using Kalman Filter
**Input:** *z_measured*—The new measurement that needs to be filtered. **Output:** *x_est*—The estimated state after applying the Kalman filter to the new measurement. **Variables:** *x_temp_est*—Temporary estimated state *P_temp*—Temporary error covariance *K*—Kalman gain *x_est*—Updated estimated state *P*—Updated error covariance *x_est_last*—Last estimated state *P_last*—Last error covariance *Q*—Process noise covariance *R*—Measurement noise covariance **Procedure** InitiateKalmanFilter: 1 Define and initialize the Kalman filter variables *Q*, *R*, *x_est_last*, *P_last* **Procedure** UpdateKalmanFilter(*z_measured*): 1 *x_temp_est* ← *x_est_last* 2 *P_temp* ← *P_last* + *Q* 3 *K* ← *P_temp* * (1.0/(*P_temp* + *R*)) 4 *x_est* ← *x_temp_est* + *K* * (*z_measured* − *x_temp_est*) 5 *P* ← (1 − *K*) * *P_temp* 6 *x_est_last* ← *x_est* 7 *P_last* ← *P* 8 **Return** *x_est* **Begin** Kalman Filter Update Procedure *filtered_value* ← UpdateKalmanFilter(z_measured) **End** Kalman Filter Update Procedure

The Equations (1)–(5) have been applied in Algorithm A3. The raw data has been noise filtering by Kalman filter before passing to MultiWii for data processing and transmission procedure as below:

**Algorithm A3** Data processing and transmission procedure using MultiWii
**Input:** *gyroData*—A structure containing gyroscope data with x, y, z attributes. *accelData*—A structure containing accelerometer data with x, y, z attributes. *magData*—A structure containing magnetometer data with x, y, z attributes. **Output:** *output*—A string that concatenates sensor data, formatted for ProcessingIDE **Procedure** ProcessDataForProcessingIDE: 1 Combine *gyroData*, *accelData*, and *magData* into a single string 2 Each sensor data value is converted to a string and separated by commas 3 Store the concatenated string in the variable output **Procedure** SendDataToProcessingIDE(output): 1 Transmit the output string to ProcessingIDE for further processing **Begin** Data Processing and Transmission Procedure *output* ← ProcessDataForProcessingIDE(*gyroData*, *accelData*, *magData*) SendDataToProcessingIDE(*output*) **End** Data Processing and Transmission Procedure

ProcessDataForProcessingIDE procedure formats the filtered sensor data from the gyroscope, accelerometer, and magnetometer into a string that is easily understood by ProcessingIDE. The data from each sensor’s x, y, and z axes are converted into strings and concatenated, with commas serving as separators. The SendDataToProcessingIDE procedure represents the action of sending the formatted data to the ProcessingIDE communicates with the controller on an I2C bus in fast mode at 400 kbit/s.

**Algorithm A4** ProcessingIDE for 3D Visualization
**Input:** *data*—Serial data stream containing IMU sensor data. **Output:** A 3D visualization of an object using IMU sensor data. **Variables:** *myPort*—Serial port object for communication. *imuDataStrings*—Array of strings to hold incoming data. *imuData*—Array of floats to store IMU data. *portName*—Name of the serial port. **Procedure** setup: 1 Initialize the 3D environment with a specified width, height, and renderer. 2 Identify and select the available serial port. 3 Open the serial port with the selected port name and specified baud rate. 4 Set the serial port to buffer until a newline character is received. **Procedure** draw: 1 Set the background of the window to blue. 2 If new IMU data are available, parse each value and update the *imuData* array. 3 Visualize the IMU data by rendering a 3D model (details of the model are not shown here). 4 Reset *imuDataStrings* to null for the next reading. **Begin** ProcessingIDE Visualization Procedure setup() // Prepare the environment and serial communication draw() // Continuous drawing loop for visualization **End** ProcessingIDE Visualization Procedure

The procedure begins with the setup phase, where the processing environment is initialized for 3D rendering, and the serial port is configured for data reception. During the main drawing loop, the program continuously updates in the background and waits for new IMU data, which upon arrival, is parsed and stored in an array. This data is then used to render a 3D model whose orientation reflects the incoming sensor data, providing a visual representation of the sensor’s position in space. The draw loop runs indefinitely, refreshing the visualization with each new set of IMU data received, effectively animating the 3D object in real-time according to the physical movements captured by the IMU.
